# Blue Light Improves Vase Life of Carnation Cut Flowers Through Its Effect on the Antioxidant Defense System

**DOI:** 10.3389/fpls.2020.00511

**Published:** 2020-05-26

**Authors:** Mostafa Aalifar, Sasan Aliniaeifard, Mostafa Arab, Mahboobeh Zare Mehrjerdi, Shirin Dianati Daylami, Margrethe Serek, Ernst Woltering, Tao Li

**Affiliations:** ^1^Photosynthesis Laboratory, Department of Horticulture, Aburaihan Campus, University of Tehran, Tehran, Iran; ^2^Faculty of Natural Sciences, Institute of Horticultural Production Systems, Floriculture, Leibniz University Hannover, Hannover, Germany; ^3^Wageningen Food & Biobased Research, Wageningen, Netherlands; ^4^Horticulture and Product Physiology, Wageningen University & Research, Wageningen, Netherlands; ^5^Institute of Environment and Sustainable Development in Agriculture, Chinese Academy of Agricultural Sciences, Beijing, China

**Keywords:** antioxidant enzymes, carnation, light spectrum, oxidative stress, radiation, vase life

## Abstract

Improving marketability and extension of vase life of cut flowers has practical significance for the development of the cut flower industry. Although considerable efforts have been made over many years to improve the vase life of cut flowers through controlling the immediate environment and through post-harvest use of floral preservatives, the impact of lighting environment on vase life has been largely overlooked. In the current study, the effect of three LED light spectra [white (400–730 nm), blue (peak at 460 nm), and red (peak at 660 nm)] at 150 μmol m^–2^ s^–1^ on vase life and on physiological and biochemical characteristics of carnation cut flowers was investigated. Exposure to blue light (BL) considerably delayed senescence and improved vase life over that of flowers exposed to red light (RL) and white light (WL). H_2_O_2_ and malondialdehyde (MDA) contents in petals gradually increased during vase life; the increase was lowest in BL-exposed flowers. As a consequence, BL-exposed flowers maintained a higher membrane stability index (MSI) compared to RL- and WL-exposed flowers. A higher activity of antioxidant enzymes [superoxide dismutase (SOD), peroxidase (POD), catalase (CAT), and ascorbate peroxidase (APX)] was detected in petals of BL-exposed flowers, compared to their activities in RL- and WL-exposed flowers. In BL-exposed flowers, the decline in petal carotenoid contents was delayed in comparison to RL- and WL-exposed flowers. Maximum quantum efficiency of photosystem II (Fv/Fm) and a higher percentage of open stomata were observed in leaves of BL-exposed flowers. Sucrose and glucose contents accumulated in petals during vase life; sugar concentrations were higher in BL-exposed flowers than in RL- and WL-exposed flowers. It is concluded that BL exposure improves the vase life of carnation cut flowers through its effect on the antioxidant defense system in petals and on photosynthetic performance in the leaves.

## Introduction

Ornamental plant production is an expanding industry worldwide and has great potential for continued future growth in international markets (Jerzy et al., 2011). However, cut flowers generally have a short vase life depending on genetic and environmental factors, and this often limits development of the industry (Kumar et al., 2014; Van Meeteren and Aliniaeifard, 2016). Carnation (*Dianthus caryophyllus* L.) is one of the most popular and important of cut flowers for the ornamental industry, also useful as an ornamental model plant, for which the genome has been sequenced (Karami et al., 2008; Yagi et al., 2013). Normally carnations have a short vase life of around 5–10 days depending on the cultivar.

Post-harvest senescence of cut flowers is an active process involving physiological and biochemical changes (Buchanan-Wollaston and Morris, 2000; Rubinstein, 2000; Battelli et al., 2011), and is regulated by a cell death program (Arora and Singh, 2006; Van Doorn and Woltering, 2008; Wagstaff et al., 2009; Van Doorn, 2011). Physiological and biochemical aspects of carnation senescence have previously been described (Sugawara et al., 2002; Shibuya and Ichimura, 2010; Satoh, 2011), and conditions during growth of mother plants, storage and handling, environment, and phyto-hormones all play roles in senescence regulation (Karimi et al., 2012; Asil et al., 2013; Hotta et al., 2016). Particularly since it is a model flower, the mechanisms involved in vase life determination have attracted much interest (Sugawara et al., 2002; Tanase et al., 2008; Satoh, 2011; Tanase et al., 2015).

In recent years considerable efforts have been made to improve vase life of cut flowers through controlling the post-harvest environment; however, the influence of lighting environment on vase life has largely been overlooked. Light is one of the most influential and versatile of environmental stimuli controlling plant life from seed germination to plant senescence (Rodrigues et al., 2014). Some studies have shown that light delays senescence during post-harvest storage of some horticultural products such as basil (Costa et al., 2013), broccoli (Zhan et al., 2012), spinach (Lester et al., 2010), mushrooms (Oms-Oliu et al., 2010), and cabbage (Perrin, 1982). However, both negative and positive effects of different light spectra on the post-harvest longevity of horticultural products such as tomato, asparagus, strawberry, broccoli, peach, lettuce, and pot *Chrysanthemum* have been reported (Jerzy et al., 2011; Dhakal and Baek, 2014; Woltering and Seifu, 2014; Xu et al., 2014; Gong et al., 2015; Hasperué et al., 2016a, b; Mastropasqua et al., 2016). To date, the effects of light spectra on post-harvest performance of cut flowers have not been studied in detail and the mechanisms underlying the responses to different spectra remain largely unknown.

Photosynthesis is the main process providing energy input for growth and development. Previous studies have reported that plants grown under blue light (BL) are characterized by higher photosynthetic electron transport and chlorophyll a/b ratios than plants grown under red light (RL) (Eskins et al., 1991). Zheng and Van Labeke (2017a,b), demonstrated that BL resulted in the highest maximum quantum efficiency (Φ_PSII_) and quantum yield (F_v_/F_m_) in *Cordyline australis*, *Ficus benjamina*, *Sinningia speciosa*, and *Chrysanthemum morifolium*. However, knowledge about the effects of the lighting environment on photosynthesis of leaves of cut flowers and the subsequent importance of photosynthetic performance on vase life is largely unknown.

Oxidative stress plays a key role in cut flower senescence. Several reports have shown that different light spectra strongly affect the activity of antioxidant systems in different plant species such as *Solanum lycopersicum* (Kim et al., 2013), *Kalanchoe pinnata* (Nascimento et al., 2013), *Fragaria ananassa* (Xu et al., 2014), *Brassica oleracea* (Deng et al., 2017), and *Camptotheca acuminata* (Yu et al., 2017). These studies revealed that an enhanced antioxidant system improves post-harvest longevity of these products (Xu et al., 2014; Deng et al., 2017). Therefore, studying the antioxidant defense system during post-harvest assessment of horticultural products will help advance our knowledge of the regulatory role of environmental cues on the vase life of cut flowers.

Only one report, Jerzy et al. (2011), studied the effects of different light spectra on post-harvest longevity of pot *Chrysanthemum*, and the literature concerning the effect of light spectra on cut flower vase life, and the underlying response mechanisms, are scarce. Therefore, the purpose of this study was to explore the effects of light spectra on the vase life of carnation flowers and to investigate the underlying mechanisms determining their vase life.

## Materials and Methods

### Plant Materials and Growth Conditions

Cut flowers of a standard type of carnation (*Dianthus caryophyllus* cv. ‘Moon light’) (40–45 cm in length) were obtained from a commercial greenhouse in the early morning. Healthy flowers were harvested at the commercial stage of flower development, described as ‘fully open,’ when the outer petals were reflected at right angles to the pedicel and the inner ones were relatively small and immature (Nichols and Ho, 1975). The stems were placed in tap water and the end of each stem was re-cut under water to 30 cm to allow for water uptake by the vessel elements of the stem. Thereafter, 12 replicates per treatment were randomly allocated to three spectral light treatments. Air temperature of the test room was set at 21 ± 2°C and flowers received a photoperiod of 12/12 h light/dark cycles.

### Light Treatments

**FIGURE 1:**
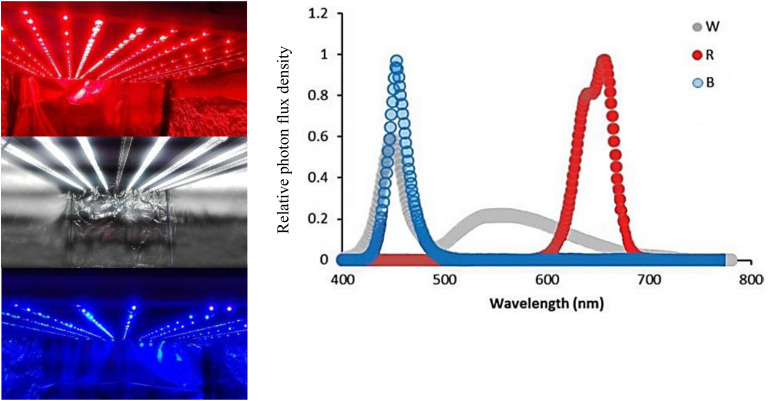
Light spectra of the white (W), red (R), and blue (B) lighting environments measured at the flower level in the cabinets.

For applying light treatments, LED modules (Iran grow light, Tehran, Iran) were placed in the top of chambers (120 cm × 90 cm × 80 cm). Three light spectra consisting of red (R; peak at 660 nm), blue (B; peak at 460 nm), and white (W; 400–730 nm) were used in the chambers without any other illumination sources. Light intensity at the flower level was set at 150 μmol m^–2^ s^–1^ by adjusting the distance of the light source. Light spectral distribution was measured using a Sekonic C7000 SpectroMaster spectrometer (Sekonic, Corp., Tokyo, Japan) in the range of 300–800 nm (Figure 1) and uniformity was verified by measuring the light intensity at five points of each light treatment at the flower level.

### Vase Life

The degree of flower wilting and vase life of flowers was determined according to the morphological stages (Paulin et al., 1986) and appearance of symptoms such as shrinkage, brown edges, wilting stems, and yellow/brown foliage (VBN, 2005). The petals were judged to have senesced when they wilted or became necrotic at the edges (Mor et al., 1980). Vase life of individual flowers was defined by the duration from the starting time to the occurrence of flower wilting (Yamamoto et al., 1992).

### Relative Fresh Weight (RFW) and Water Uptake

Flower stems were daily weighed and the following formula was used to measure relative fresh weight:

(1) RFW(%)=(Freshweightofflowerduringvasestage-Freshweightoffloweratday 0)Freshweightoffloweratday 0×100

Flower weight was determined at time 0 and at 2, 4, 6, 8, 10, 12, and 15 days following onset of the trial (Zeng et al., 2011). Flowers were placed in 300 mL of tap water. At the different time points, the amount of water (mL) taken up was measured and thereafter the level was brought back up again to 300 mL. At the end of the trial, cumulative water uptake was calculated.

### Malondialdehyde (MDA) and Hydrogen Peroxide (H_2_O_2_) Contents

Malondialdehyde (MDA) and H_2_O_2_ were measured as indices of lipid peroxidation and oxidative stress. The petals of flowers were collected and ground in liquid nitrogen and then homogenized in 4% ice-cold trichloroacetic acid (TCA). The homogeneous samples were then centrifuged at 13,000 *g* for 15 min at 5°C. The supernatants were used for the measurement of MDA (Heath and Parker, 1968) and H_2_O_2_ (Guo et al., 2006) contents. Absorbance was spectrophotometrically (Lambda 25 UV/VIS) measured at 532 and 500 nm for H_2_O_2_ and MDA, respectively. The MDA and H_2_O_2_ were expressed as μmol g^–1^ on a fresh weight basis.

### Petal Membrane Stability Index (MSI)

For determining petal membrane stability, two samples of plant material (15 disks of petals) were prepared and dipped in 10 mL double distilled water. One of them was kept at 40°C for 30 min and its electrical conductivity (EC) was measured (EC1). The second sample was kept in a boiling water bath (100°C) for 10 min, cooled to room temperature, and EC also recorded (EC2). MSI was determined according to Sairam et al. (1997) based on the following equation:

$$\text{MSI}\left(\%\right)=\left[1-\left(\frac{\mathrm{EC1}}{\mathrm{EC2}}\right)\right]\times 100$$

### Measurement of Carbohydrate Levels

Samples from petals were collected during the vase life of flowers (0, 8, 10, and 15 days following start of the trial) and immediately frozen in liquid nitrogen then stored at −80°C. Sugars (sucrose and glucose) and starch were determined by enzymatic assays according to Trethewey et al. (1998) and Hajirezaei et al. (2000). Concentrations of sucrose and glucose were determined in microplates via NADH-specific extinction at 340 nm. The results were expressed as μmol g^–1^ on a fresh weight basis.

### Pigment Analysis

Carotenoids were determined by extraction in 80% acetone as described by Lichtenthaler and Buschmann (2001). Their absorbance was recorded at 470 nm with a spectrophotometer (Lambda 25 UV/VIS) according to Lichtenthaler and Buschmann (2001). The entire process was conducted in low light conditions and placing the samples on ice, and the results expressed as mg g^–1^ on a fresh weight basis.

### Determination of Antioxidant Enzyme Activity

Frozen petal samples (0.2 g for each replicate) were ground in 5 mL of 50 mM sodium phosphate-buffer (pH 7.8) at 4°C. Samples were centrifuged at 13,000 *g* for 20 min and the supernatant was used to measure the activity of antioxidant enzymes. The 3-mL reaction solution for SOD contained 63 μM ρ-nitro blue tetrazolium chloride, 1.3 μM riboflavin, 13 μM methionine, 50 mM phosphate buffer (pH 7.8), and enzyme extract. Absorbance was measured at 560 nm with a spectrophotometer (Lambda 25 UV/VIS). The 3-mL reaction solution for CAT contained 50 mM phosphate buffer (pH 7.0), 15 mM H_2_O_2_, and 50 μl of enzyme extract. The reaction was initiated by adding enzyme extract. The decrease of absorbance of H_2_O_2_ over 1 min at 240 nm was recorded. The 3-mL reaction solution for APX contained 0.5 mM AsA, 0.1 mM H_2_O_2_, 50 mM phosphate buffer (pH 7.0) and 0.1 mL enzyme extract. APX activity was evaluated by following the decrease in absorbance of AsA over 1 min at 290 nm. The 3-mL reaction solution for POX contained 0.2% (w/v) *o*-dianisidine, 0.1M potassium phosphate buffer (pH 7.8) and the enzyme extract. Absorbance was measured at 470 nm (Han et al., 2008; Chen et al., 2009). The results were expressed as μmol min^–1^ mg^–1^ of total protein for CAT, APX and POD and U mg^–1^ for SOD.

### Stomatal Opening and Polyphasic Chlorophyll a Florescence (OJIP)

On the 8th day of vase life at 2 h after exposure to different light treatments, stomatal opening was determined using a nail polish replica method on the lower epidermis of the second lateral leaflets from the apex (abaxial side) as described by Aliniaeifard and Van Meeteren (2016). The nail polish layer was separated with a strip of transparent sticky tape and pasted on a glass slide and observed using a light microscope (BH-323, Olympus, Tokyo, Japan). Pore aperture of more than 6 μm was considered as open stomata, less than 3 μm as closed, and between 3 and 6 μm as semi-closed. The polyphasic chlorophyll a fluorescence (OJIP) transients were determined using a Fluorpen FP 100-MAX (Photon Systems Instruments, Drásov, Czechia) on young fully expanded carnation leaves after 20 min dark adaptation (Strasser, and Strasser, 1995) according to the JIP test (Strasser et al., 2000) at 1, 4, 8, and 12 days following onset of the trial. The measurement of transient fluorescence was induced by a saturating light of 3000 μmol m^–2^ s^–1^ PPFD. Three leaves of each cut carnation stem were used for each replicate. The parameters obtained from this protocol were calculated according to Kalhor et al. (2018).

### Statistical Analysis

The data were statistically evaluated by the ‘completely randomized design’ method using SAS software (Statistical Analysis System, Version 9). Means were separated using the Duncan Multiple Range test at a significance level of 0.01. Three (for all measurements traits except vase life) or nine (vase life) replicates were considered for each treatment. A pooled sample of outer and inner petals from each cut carnation flowers was used for measurements of biochemical traits for each replicate. In Figures 3–8, the measurements were conducted up until the end of vase life.

## Results

### Blue Light Improves the Vase Life of Carnation Cut Flowers

**FIGURE 2:**
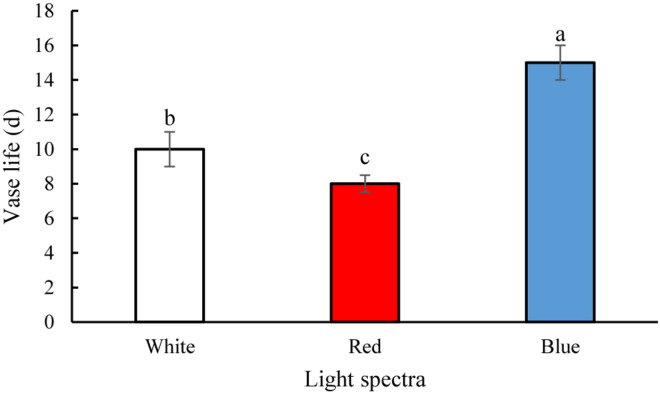
Effects of light spectra on the vase life of carnation cut flowers. Averages ± SEM (*n* = 9) are shown. Cut flowers were placed in cabinets (120 cm × 90 cm × 80 cm) with different light spectra (white, red, or blue light). Light intensity at the flower level was set at 150 μmol m^–2^ s^–1^. Air temperature of the test room was set at 21 ± 2°C and flowers received a photoperiod of 12/12 h light/dark cycles. Different letters indicate that values are significantly different at *P* < 0.01 according to Duncan’s multiple range tests.

**FIGURE 3:**
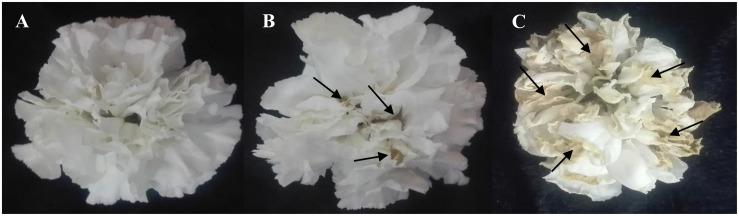
Effects of light spectra (**A**; blue light, **B**; white light, and **C**; red light) on vase life of carnation cut flowers on the 10th day of vase life. Cut flowers were placed in cabinets (120 cm × 90 cm × 80 cm) with different light spectra (white, red, or blue light). Light intensity at the flower level was set at 150 μmol m^–2^ s^–1^. Air temperature of the test room was set at 21 ± 2°C and flowers received a photoperiod of 12/12 h light/dark cycles. The arrows indicate areas that are turning yellow and brown.

The vase life of flowers was significantly (*P* > 0.01) prolonged by exposure to B spectral light. The vase life of BL-exposed flowers was 5 days longer than the vase life of WL-exposed flowers (Figures 2, 3). Exposure to RL shortened the vase life of flowers in comparison with WL exposure (Figures 2, 3). Exposure to BL approximately doubled the vase life compared to RL exposure (Figures 2, 3). These results are indicative of positive effects of B wavelength on the post-harvest performance of carnation flowers.

### Relative Fresh Weight (RFW) and Water Uptake of Cut Flowers

**FIGURE 4:**
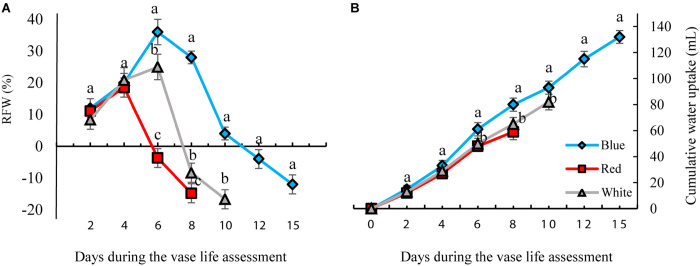
Effects of different light spectra (blue, red, and white) on the relative fresh weight [RFW, **(A)**] and cumulative water uptake **(B)** of carnation cut flowers during storage. Cut flowers were placed in cabinets (120 cm × 90 cm × 80 cm) with different light spectra (white, red, or blue light). Light intensity at the flower level was set at 150 μmol m^–2^ s^–1^. Air temperature of the test room was set at 21 ± 2°C and flowers received a photoperiod of 12/12 h light/dark cycles. Averages ± SEM (*n* = 3) are presented. Different letters indicate that values are significantly different at *P* < 0.01 according to Duncan’s multiple range tests.

The RFW of flowers increased till day 4 of vase life in RL-exposed flowers and till day 6 of BL- and WL-exposed flowers, and decreased thereafter (Figure 4). There was no distinct difference in RFW among the light treatments during the first 4 days. On the 6th day of vase life, the RFW of RL-exposed flowers was three and four times lower than that of WL- and BL-exposed flowers. Following day 6 till the end of vase life assessment, RFW of BL-exposed flowers was significantly higher than the RFW of flowers exposed to other light spectra (Figure 4).

The highest and lowest water uptake was found in BL- and RL-exposed flowers, respectively. At the end of these measurements, the cumulative water uptake of BL-exposed flowers was 30 and 18% more than that of RL- and WL-exposed flowers. These results indicate that exposure to BL significantly improves water uptake and postpones weight loss of flowers during their vase life.

### Blue Light Delays and Reduces Severity of Oxidative Damage to Carnation Petals

**FIGURE 5:**
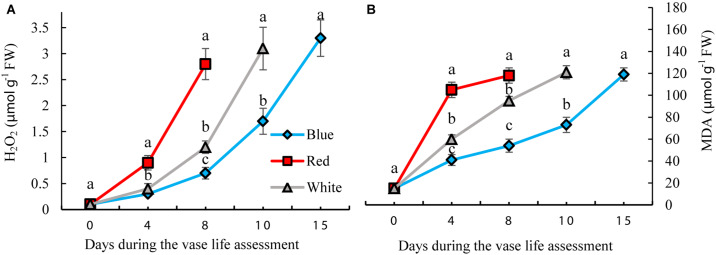
Effects of different light spectra on H_2_O_2_ **(A)** and Malondialdehyde (MDA) **(B)** contents in the petals of carnation cut flowers during vase life. Cut flowers were placed in cabinets (120 cm × 90 cm × 80 cm) with different light spectra (white, red, or blue light). Light intensity at the flower level was set at 150 μmol m^–2^ s^–1^. Air temperature of the test room was set at 21 ± 2°CC and flowers received a photoperiod of 12/12 h light/dark cycles. Means ± SEM are presented (*n* = 3). Different letters indicate that values are significantly different at *P* < 0.01 according to Duncan’s multiple range tests.

H_2_O_2_ and MDA contents were measured to assess the oxidative damage to the petals. H_2_O_2_ gradually accumulated in the carnation petals during vase life. BL significantly reduced the concentration of H_2_O_2_ by 35, 25, and 42% on days 4, 8, and 10, respectively, compared to the concentration of H_2_O_2_ in WL-exposed flowers (Figure 5A). In petals of RL-exposed flowers, concentrations of H_2_O_2_ showed an earlier increase than with the other light spectra. On the 8th day of vase life, H_2_O_2_ content in RL-exposed flowers was four times higher than in BL-exposed flowers. Similar to H_2_O_2_, MDA content also gradually accumulated in the carnation petals during vase life. Exposure to BL caused reductions in MDA contents during the whole vase life assessment period compared to the MDA content of RL- and WL-exposed flowers. MDA content in RL-exposed flowers was approximately doubled in comparison with BL-exposed flowers during vase life (Figure 5B). These results indicate that post-harvest application of BL can decrease the severity of oxidative damage to carnation petals.

### Blue Light Enhances Antioxidant Enzyme Activities in the Petals of Carnation Cut Flowers

**FIGURE 6:**
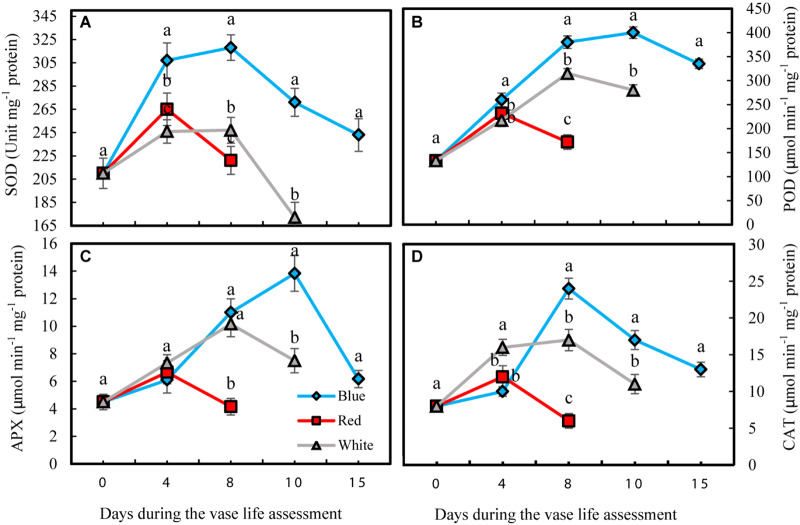
Effects of light spectra on the antioxidant enzymes activity during vase life in cut carnation flower petals. Cut flowers were placed in cabinets (120 cm × 90 cm × 80 cm) with different light spectra (white, red, or blue light). Light intensity at the flower level was set at 150 μmol m^–2^ s^–1^. Air temperature of the test room was set at 21 ± 2°C and flowers received a photoperiod of 12/12 h light/dark cycles. **(A)** Superoxide dismutase (SOD); **(B)** Peroxidase (POD); **(C)** Ascorbate peroxidase (APX), and **(D)** Catalase (CAT). Means ± SEM are presented (*n* = 3). Different letters indicate that values are significantly different at *P* < 0.01 according to Duncan’s multiple range tests.

Antioxidant enzyme activities in petals were significantly influenced by light spectra during vase life. Activity of antioxidant enzymes was divided into two phases for all the flowers that were exposed to different light spectra; in the first phase there was an induction in the activity of antioxidant enzymes, and in the second phase their activities declined (Figure 6). For the RL-exposed flowers, activities of antioxidant enzymes reached their maximum levels at an earlier time than the BL- and WL-exposed flowers. The magnitude of increase in antioxidant enzyme activities in BL-exposed flowers was significantly greater than the amplification of enzyme activity in RL- and WL-exposed flowers. In the case of SOD, its activity in BL-exposed flowers was always higher than its activity in RL- and WL-exposed flowers following the start of vase life (Figure 6A). After 4 days, POD activity in BL-exposed flowers was higher than in RL- and WL-exposed flowers (Figure 6B). APX and CAT activities in RL-exposed flowers at the last day of vase life were lower than initial activities, while the activity of these enzymes were more than doubled or tripled in their maximum activities in WL- and BL-exposed flowers, respectively (Figures 6C,D). These results indicate that BL is an environmental signal that results in augmentation of petal antioxidant enzyme activities.

### Blue Light Maintains Membrane Stability Index (MSI) During Vase Life of Carnation

**FIGURE 7:**
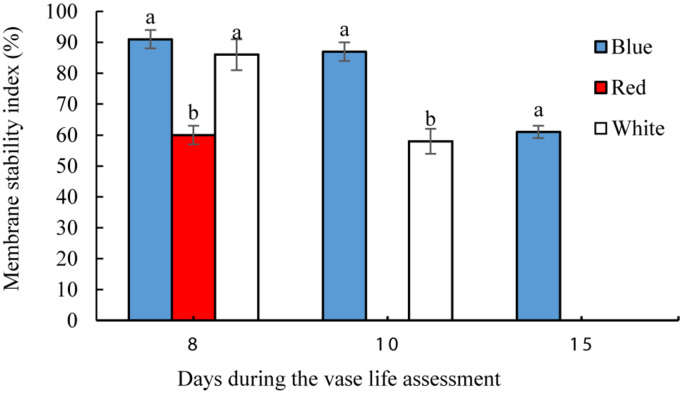
Effects of light spectra on petal membrane stability index (MSI) of carnation cut flowers during vase life. Cut flowers were placed in cabinets (120 cm × 90 cm × 80 cm) with different light spectra (white, red, or blue light). Light intensity at the flower level was set at 150 μmol m^–2^ s^–1^. Air temperature of the test room was set at 21 ± 2°C and flowers received a photoperiod of 12/12 h light/dark cycles. Means ± SEM are presented (*n* = 3). Different letters indicate that values are significantly different at *P* < 0.01 according to Duncan’s multiple range tests.

On the 8th day of vase life assessment, BL- and WL-exposed flowers had significantly (*P* < 0.01) higher petal MSI than in the petals of RL-exposed flowers (Figure 7). On the 10th day, the highest MSI (87%) was detected in BL-exposed flowers, significantly higher than the MSI of WL-exposed flowers (58%). On the 15th day when petals of RL- and WL-exposed flowers were wilted, BL-exposed flowers retained 61% MSI in their petals (Figure 7). These results indicate that BL maintains the stability of the petals during storage and delays senescence.

### Blue Light and Duration of Storage Increase Petal Carbohydrate Content

Carbohydrate status of the petals was largely influenced by exposure of flowers to different light spectra. Concentrations of sucrose and glucose and starch were determined during the vase life in the petals of cut carnation flowers under different light spectra. The starch level in the petals invariably remained below the detection limit of 0.5 μmol g^–1^ FW (data not shown).

**FIGURE 8:**
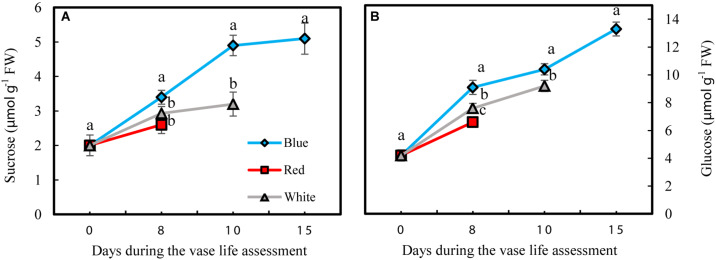
Effects of different light spectra on the content of sucrose **(A)** and glucose **(B)** in the petals of carnation cut flowers during vase life. Cut flowers were placed in cabinets (120 cm × 90 cm × 80 cm) with different light spectra (white, red, or blue light). Light intensity at the flower level was set at 150 μmol m^–2^ s^–1^. Air temperature of the test room was set at 21 ± 2°C and flowers received a photoperiod of 12/12 h light/dark cycles. Means ± SEM are presented (*n* = 3). Different letters indicate that values are significantly different at *P* < 0.01 according to Duncan’s multiple range tests.

Petals had lower sucrose levels compared to glucose under all different light spectra (Figure 8). Concentrations of both glucose and sucrose increased over the whole vase life period in flowers under all light spectra (Figures 8A,B). Concentrations of both glucose and sucrose were higher in the petals of BL-exposed flowers in comparison with their concentrations in WL and RL-exposed flowers during all stages of vase life (Figures 8A,B). At day 15, sugar levels in petals of BL-exposed flowers were 2.5 times more than their initial levels and were doubled compared to their levels in RL-exposed flowers on the last day of their vase life (8 and 10 days from onset of experiment).

### Effect of Light Spectra on the Concentration of Carotenoids in Flowers

**FIGURE 9:**
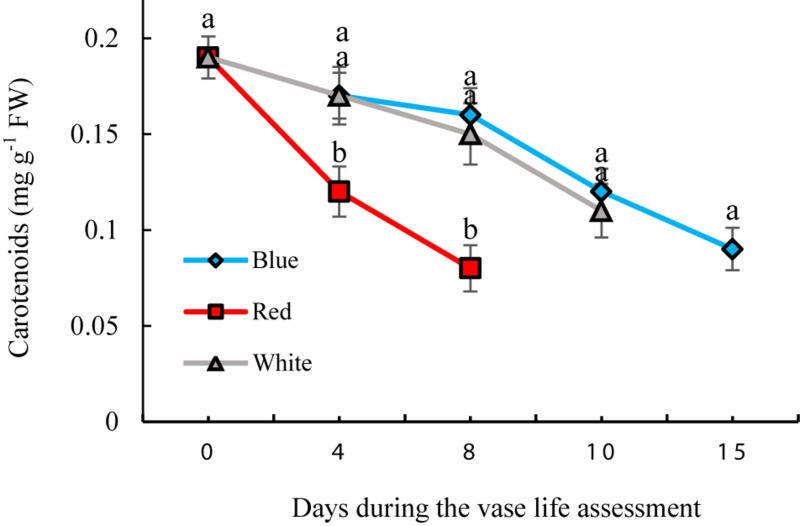
Effects of different light spectra on the content of carotenoids in cut carnation flower. Cut flowers were placed in cabinets (120 cm × 90 cm × 80 cm) with different light spectra (white, red, or blue light). Light intensity at the flower level was set at 150 μmol m^–2^ s^–1^. Air temperature of the test room was set at 21 ± 2°C and flowers received a photoperiod of 12/12 h light/dark cycles. Means ± SEM are presented (*n* = 3). Different letters indicate that values are significantly different at *P* < 0.01 according to Duncan’s multiple range tests.

Significant differences were observed in concentrations of carotenoids during the vase life of flowers exposed to different light spectra. The concentrations gradually decreased during the vase life in all treatments; however the extent of this decline was highest in RL-exposed flowers (Figure 9). Under exposure to BL and WL, the decreases in carotenoid contents were less pronounced compared to their contents under RL (Figure 9). These results showed the negative role of RL in maintaining carotenoids of carnation petals.

### Stomatal Status and Photosynthetic Biophysical Analysis in Leaves

Stomatal status in the leaves of carnation cut flowers was affected by light spectra (Table 1). A higher percentage of fully open stomata was observed on the leaves of BL-exposed flowers (95%) than in the RL-exposed flowers (0%) (Table 1). WL-exposed flowers showed a high percentage of semi-closed stomata (80%) (Table 1).

**TABLE 1:** Percentage of open, closed, and semi-closed stomata in the leaves of carnation cut flowers following 8 days of exposure to different light spectra.

Light spectra	Stomatal status		
	Open	Closed	Semi-closed
Blue	95^*a*^	0^*c*^	5^*b*^
White	5^*b*^	15^*b*^	80^*a*^
Red	0^*c*^	100^*a*^	0^*c*^

*Pore aperture more than 6 μm was considered as open stomata, less than 3 μm as closed, and between 3 and 6 μm as semi-closed. Twenty stomata (n = 20) from three leaves were considered for each replicate. Sampling was done at 2 h after exposure to different light treatments. Letters indicate that values significantly different at P < 0.01 according to Duncan’s multiple range tests.*

**FIGURE 10:**
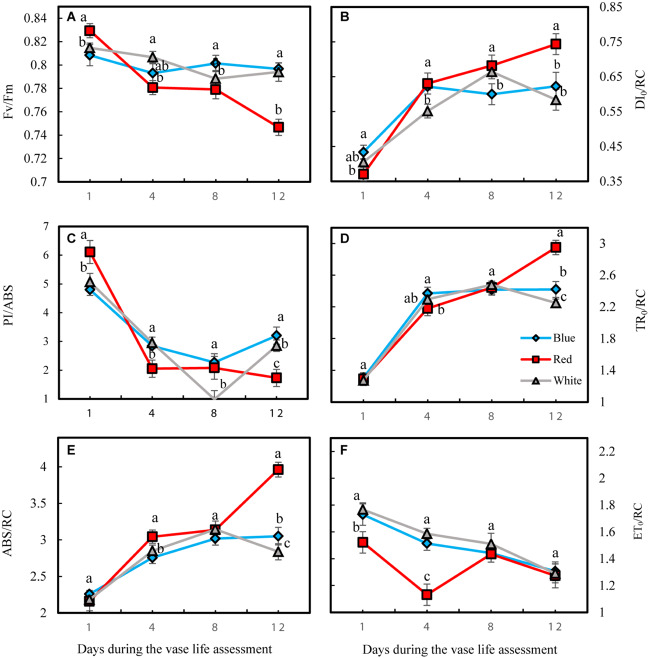
Parameters related to OJIP test including Fv/Fm **(A)**, DI_0_/RC **(B)**, PI/ABS **(C)**, TR_0_/RC **(D)**, ABS/RC **(E)**, and ET_0_/RC **(F)** obtained from the transient florescence exhibited by leaves of carnation. Cut flowers were placed in cabinets (120 cm × 90 cm × 80 cm) with different light spectra (white, red, or blue light). Light intensity at the flower level was set at 150 μmol m^–2^ s^–1^. Air temperature of the test room was set at 21 ± 2°C and flowers received a photoperiod of 12/12 h light/dark cycles. Means ± SEM are presented (*n* = 3). Different letters indicate that values are significantly different at *P* < 0.01 according to Duncan’s multiple range tests.

At the 1st day of the vase life, there was no difference among Fv/Fm in leaves of flowers under different light spectra (Figure 10A). Fv/Fm of RL-exposed flowers significantly decreased during vase life, while no significant difference was observed for Fv/Fm of BL- and WL-exposed flowers. At day 12 of vase life, the highest and lowest F_v_/F_m_ was detected in BL- and RL-exposed flowers, respectively (Figure 10A). The specific energy fluxes per reaction center (RC) for energy absorption (ABS/RC), dissipated energy flux (DI_0_/RC) and trapped energy flux (TR_0_/RC) increased but performance index on the absorption basis (PI/ABS) and electron transport flux (ET_0_/RC) decreased during vase life for flowers under all light spectra (Figure 10). At day 12, highest ABS/RC, DI_0_/RC and TR_0_/RC and lowest PI/ABS and ET_0_/RC were detected in RL-exposed flowers, while no significant difference was detected for these parameters between BL- and WL-exposed flowers (Figure 10). These results are indicative of the negative impact of RL on the photosynthetic performance of carnation leaves.

## Discussion

Petal senescence is a major physiological process that influences quality attributes and limits vase life of cut flowers (Kumar et al., 2014). Post-harvest application of light spectra on vegetable and fruit has attracted much attention in recent times (Dhakal and Baek, 2014; Gong et al., 2015; Hasperué et al., 2016a, b), with some studies showing beneficial effects of light on vase life of hyacinth (Krzymińska and Karasiewicz, 2017) and pot chrysanthemums (Jerzy et al., 2011). Information regarding the role of different light spectra on vase life of cut flowers is scarce however. In the present study, we exposed carnation cut flowers during vase life to 150 μmol m^–2^ s^–1^ of white, blue or red light derived from LEDs for 12 h per day. BL significantly delayed flower senescence and therefore extended vase life.

Senescence of petals in cut flowers is related to a series of highly regulated biochemical and physiological processes. The maintenance of water relations and good water uptake are among the most important factors for the vase life of a variety of cut flowers (Slootwet, 1995; Van Meeteren and Aliniaeifard, 2016). Adverse water relations can lead to problems in flower opening, premature petal wilting and bending of the pedicel, all resulting in shortened vase life (Yamada et al., 2007; Aliniaeifard and Van Meeteren, 2016). It is well-recognized that light spectra are involved in stomatal movement and that BL stimulates stomatal opening (Noichinda et al., 2007). Therefore BL might increase water transport efficiency by increasing transpiration rate and water uptake. Unlike vegetables and fruit (Xu et al., 2014; Hasperué et al., 2016b; Van Meeteren and Aliniaeifard, 2016), elevated water uptake, higher transpiration rates and water loss are beneficial for cut flowers placed in water (Elibox and Umaharan, 2010; Lu et al., 2010). Findings of the present study confirmed that the openness of stomata in the BL treatment coincided with an improved vase life of flowers, increased water uptake, transpiration rate and fresh weight.

Blue light improved carbohydrate levels in the petals during vase life (Figure 6). Sugars are the major source of energy and their deficiency results in senescence of cut flowers (Liao et al., 2000; Singh et al., 2008). Sucrose and glucose are involved in diverse plant processes including antioxidant metabolism, anthocyanin biosynthesis and storage and cell wall biosynthesis. It has been reported that higher amount of sugars in petals is associated with a delay in the senescence of cut flowers (Solfanelli et al., 2006; Agulló-Antón et al., 2011). The starch content in the carnation petals was very low (below the detection limit of 0.5 μmol g^–1^ FW); similar results have been obtained for the amount of starch in carnation by Agulló-Antón et al. (2011). Change in sugar levels as a result of exposure to different light spectra has been previously reported (Ohashi-Kaneko et al., 2007; Mastropasqua et al., 2016). Light is the driving force of photosynthesis for production of carbohydrates. Influence of light spectrum on the photosynthetic performance of flowers such as roses during cultivation has been recently reported (Bayat et al., 2018). Consistent with our results, Miao et al. (2019) reported that application of BL induced better photosynthetic performance than RL in cucumber plants during cultivation. Similar results were also reported for *Chrysanthemum*, *Cordyline australis*, *Ficus benjamina*, and *Sinningia speciosa* during cultivation (Zheng and Van Labeke, 2017a, b). In the current study in carnation, BL (and also WL) maintained a high performance of photosynthesis through limiting energy dissipation and elevating electron transport in the electron transport chain of the photosynthetic apparatus. This consequently resulted in an increase in sugar content from harvest to senescence. Application of ^14^C-sucrose showed that carnation petals act as a strong sink for absorption of sucrose (Ho and Nichols, 1975; Nichols and Ho, 1975). The significant increase in the sugar content in petals under BL might be correlated with the preserved photosynthetic ability in the leaves (Hogewoning et al., 2010; Muneer et al., 2014; Zheng and Van Labeke, 2017a, b) and translocation of sugars from the leaves toward the petals (Ho and Nichols, 1975), where a significant increase in the two sugars occurred. Nichols and Ho (1975) showed that the sucrose levels decrease during flower senescence in carnation. Decrease in soluble carbohydrate levels is related to lack of a carbohydrate source for cut flowers (Van Geest et al., 2016). It has been reported that carbohydrate levels decrease during vase life of different genotypes of chrysanthemum. However, when the flowers are fed by sucrose the levels of glucose and fructose increase during vase life (Van Geest et al., 2016). In the present experiment, due to presence of light intensities above the light compensation point, photosynthesis in leaves and calyx may have provided sugars for the flowers (as the main sink of carbohydrates). As a result it led to elevation in glucose and fructose levels during the vase life. The decrease in carbohydrate content in carnation reported by Ho and Nichols (1975) was probably due to light levels below the light compensation point during the vase life study. This indicates that the performance of the photosynthesis system plays an important role in extending vase life of carnation flowers. However, whether photosynthesis-derived carbohydrates directly influence the vase life of cut flowers is a matter for further research.

Oxidative stress was decreased and the antioxidant defense system was augmented in petals as a result of exposure to BL (Figures 4–6). The carotenoids are among the most important pigments that take part in antioxidative defense in higher plants (Yu et al., 2017). In the present study, the concentration of carotenoids decreased in flowers under all light spectra, but BL maintained higher carotenoids during vase life (Figure 9). Similar results, where application of BL increased the concentration of carotenoids, have been found in lettuce, pepper and broccoli (Li and Kubota, 2009; Gangadhar et al., 2012; Kopsell and Sams, 2013; Hasperué et al., 2016a). These results are similar to those reported by Hasperué et al. (2016a,b); who reported elevated carotenoid levels as a result of BL application as a post-harvest treatment on brussels sprouts and broccoli. It seems that stability and presence of appropriate levels of carotenoids is important to maintain the integrity of membranes, ROS detoxification and other associated physiological processes. Damage to membranes and H_2_O_2_ accumulation are considered as oxidative damage during senescence processes (Dhindsa et al., 1981; Bayat et al., 2018), and previous studies have shown that ROS accumulation is coincident with flower senescence (Rogers, 2013). Antioxidant enzymes such as SOD, CAT, APX and POD are the most important defense enzymes for detoxification of ROS in plant tissues (Ye et al., 2017). Increase in antioxidant enzymes activity usually helps neutralize the potential harmful effects of photo-oxidative damage to tissues (Lee et al., 2016; Hasan et al., 2017). Elevated antioxidant enzyme activity, and as a consequence, decrease in lipid peroxidation and H_2_O_2_ content, have been reported in response to BL in vegetables such as lettuce (Johkan et al., 2010), tomato (Kim et al., 2013), Chinese cabbage and kale (Lee et al., 2016). In the present study, SOD and CAT activities were elevated as a result of exposure to BL. Ye et al. (2017) reported higher activity of SOD and CAT in *Anoectochilus roxburghii* leaves with exposure to BL than with exposure to RL. Similar results were also reported for *Rehmannia glutinosa* (Manivannan et al., 2015) and tomato plants (Kim et al., 2013). APX and CAT in the AsA-GSH cycle and also POD enzyme are responsible for the degradation of H_2_O_2_ generated by SOD in plant cells. We showed that the activity of CAT, APX, and POD had similar patterns of change to that observed for SOD activity in BL-exposed flowers, with higher APX, SOD, POD, and CAT activities during vase life; as a result, they contained lower H_2_O_2_ in their petals in comparison with RL and WL treatments. This is in line with the findings of Manivannan et al. (2015) and Simlat et al. (2016) who also showed that BL induces activation of the antioxidant defense system in *Rehmannia glutinosa* and *Stevia rebaudiana*. These findings suggest that CAT, APX and POD work in a coordinated manner to scavenge H_2_O_2_ and that BL might induce their activities. MDA is the product of membrane lipid peroxidation, which is indicative of compromised integrity and structure of the membranes. In line with the other ameliorative effects of BL on oxidative damage, it delayed MDA accumulation in the petals and improved MSI compared to MDA and MSI under other light spectra. This resulted in a decrease in lipid degradation and peroxidation of cell membranes. These results are in accordance with those reported by Xu et al. (2014), who found a postponing effect of BL on MDA content of strawberry fruit during storage.

Of the parameters that were influenced by the light treatments the greatest difference was observed between BL and RL, WL being intermediate or similar to BL. This may be related to the relatively high proportion of B spectrum (41% in the range of 400–500 nm and 18% in the range of 600–700 nm) in the white LEDs. The observed beneficial effects of BL and to a lesser extent WL on, for example, vase life and the antioxidant enzymes, presumably are related to the percentage of BL. However, one could also argue that the lower proportion of RL caused the observed effects.

Carnation vase life is under the influence of ethylene. The longer vase life observed in our experiments under BL may have been due to lower ethylene production, although this was not measured. Light levels and quality can influence ethylene production, but the exact mechanism is still poorly understood. In addition, depending on the tissue under study and the other environmental conditions, light and different wavelengths may either stimulate or reduce ethylene production. There is currently no information available on the effect of light on ethylene production and its signaling pathway in cut flowers, and this would be an interesting topic for further experiments.

## Conclusion

The most important finding of the present study is that BL has a positive effect on the post-harvest life of carnation at physiological and biochemical levels. Our report on physiological and biochemical changes during vase life of flowers under different light spectra provides valuable information regarding the mechanism underlying the beneficial effects of BL on the determination of vase life. Therefore, management of lighting during post-harvest storage is important for improving vase life of cut carnation flowers. Based on the obtained results, BL boosted the antioxidant defense system in carnation cut flowers. In our experiments, cut flowers were exposed during their vase life to light levels that are well above the levels applied in the consumer’s home. Therefore, application of the results would not likely be with the consumer. This intensity of light, however, can be applied during storage and in retail facilities. As well as lower temperatures, a novel display design including LED light of suitable wavelengths and intensity may significantly prolong the storage or display life of the flowers and may serve as an non-chemical flower preservative.

## Data Availability Statement

The datasets generated for this study are available on request to the corresponding author.

## Author Contributions

MAa, SA, MAr, and MM planned and designed the research, performed the experiments, analyzed the data, and wrote the final version of the manuscript. MS, SD, and EW critically revised the manuscript and edited it to present form. TL helped in providing the materials and critically revised the manuscript. All authors read and approved the final manuscript.

## Conflict of Interest

The authors declare that the research was conducted in the absence of any commercial or financial relationships that could be construed as a potential conflict of interest.
